# Mitochondrial haplogroup M contributes to asthma risk in the Kuwaiti population

**DOI:** 10.3389/falgy.2025.1618964

**Published:** 2025-08-08

**Authors:** Mohammed Dashti, Hussain Bahbahani, Hussain Alsaleh, Thangavel Alphonse Thanaraj, Fahd Al-Mulla

**Affiliations:** ^1^ Translation Research Department, Dasman Diabetes Institute, Kuwait City, Kuwait; ^2^ Department of Biological Sciences, Faculty of Science, Kuwait University, Al-Shidadya, Kuwait; ^3^ Saad Al-Abdullah Academy for Security Sciences, Ministry of Interior, Kuwait City, Kuwait

**Keywords:** mitochondrial haplogroups, asthma genetics, Kuwaiti population, mitochondrial DNA variants, haplogroup M

## Abstract

**Background:**

Asthma is a multifactorial chronic inflammatory disease characterized by intermittent airflow obstruction, which may result in irreversible pathological remodelling of the airways. In Kuwait, the prevalence of asthma among young adults is approximately 11%, with a strong maternal influence on asthma risk. While nuclear genetic studies have identified several asthma-associated loci, the role of maternally inherited mitochondrial DNA (mtDNA) in asthma susceptibility remains poorly understood, particularly in Middle Eastern populations.

**Methods:**

In this exploratory study, we analysed mtDNA from 287 Kuwaiti individuals, including 48 asthmatics and 239 controls, extracted from whole-exome sequencing data (average coverage 27×), with variant calling via GATK and haplogroup assignment using HaploGrep2. Logistic regression was used to assess associations between mtDNA variants/haplogroups and asthma, adjusting for age, sex, and BMI.

**Results:**

Mitochondrial haplogroup M was identified as a significant risk factor for asthma (OR = 3.37; 95% CI = 1.09–10.42; *P* = 0.035). Additionally, we identified fourteen mtDNA variants associated with asthma risk through complementary case-control and exclusivity analyses. These variants are located within genes encoding subunits of mitochondrial Complex I (*MT-ND1*, *MT-ND3*, *MT-ND5*), Complex III (*MT-CYB*), Complex IV (*MT-CO1*, *MT-CO2*), and the mitochondrial control region. Most are linked to dysfunction and reactive oxygen species (ROS) production, key processes implicated in asthma pathogenesis.

**Conclusions:**

Our findings suggest that mitochondrial haplogroup M and specific mtDNA variants contribute to asthma susceptibility in the Kuwaiti population. These insights provide a foundation for future research on mitochondrial genetic influences in asthma and highlight the need for larger studies to validate these associations and explore potential therapeutic implications.

## Introduction

Asthma is a chronic inflammatory disorder of the lungs, specifically affecting the bronchi and bronchioles. It is characterized by intermittent airflow obstruction, airway hyper-responsiveness, and recurrent episodes of breathlessness, wheezing, chest tightness, and coughing. If left untreated, it can lead to irreversible airway remodelling and intractable airflow limitation (1, 2). Asthma represents a major global health concern, affecting an estimated 262 million individuals worldwide, and its prevalence continues to increase (3).

In Kuwait, asthma prevalence is notably high, with recent estimates between 11%–15% among adults, 18% in children, and an overall adjusted rate of approximately 9.5%—the highest in the Gulf region (4, 5). Asthma-related hospital admissions and mortality also remain significant. Although hospitalization rates declined by 49.5% and mortality by 77.6% from 2000 to 2014 (6), recent WHO data (7) reported an age-adjusted mortality rate of 1.12 per 100,000, reflecting a persistent disease burden despite improved management. Kuwaiti nationals experience significantly higher risks than non-Kuwaitis for both hospitalizations and mortality, as indicated by adjusted rate ratios exceeding 1.8 and 1.2, respectively. This disparity arises from environmental factors (e.g., dust storms, historical oil-related pollution, humidity, indoor allergens like incense), behavioural risks (e.g., smoking prevalence of 20%–30%, household second-hand smoke), socioeconomic issues (e.g., stigma, delayed care, emergency service overuse), and clinical factors (e.g., high comorbidity rates such as allergic rhinitis in 70%–80%, obesity around 40%, and poor adherence to medications) (6, 8). Non-Kuwaitis, often expatriates, may be underdiagnosed or repatriated in severe cases, artificially reducing reported rates (6). Psychological stressors, including war-related trauma, further increase risks among Kuwaitis (9). Compared to other populations, Kuwait's asthma prevalence (9.5%–18%) is generally lower than Caucasian cohorts (10%–20%) but higher than East African populations (∼5%–6%). Differences are likely due to varying environmental exposures and genetic factors (5, 10). Kuwait's unique context—with high consanguinity rates (∼50%) increasing the expression of recessive genetic variants in key immune pathways and significant environmental stressors—exacerbates asthma susceptibility, severity, and allergic phenotypes, highlighting the need for targeted research and interventions (8, 11).

Asthma is a multifactorial disorder where genetic and environmental factors interact to influence disease susceptibility and severity (12). Numerous genome-wide association studies (GWAS) have identified key autosomal genetic loci of asthma, including genes involved in immune response and inflammation pathways, such as *TSLP*, *TNFSF4*, *ADORA1*, *CHIT1*, *GATA3*, and *MUC5AC* (1, 2, 13, 14). These genes primarily regulate immune responses, epithelial barrier function, and inflammatory processes associated with asthma pathogenesis. However, despite these insights, a parental history of allergy—particularly from the maternal side—remains a notably strong predisposing factor (15–18). Children of asthmatic mothers are at higher risk of developing asthma compared to those with non-asthmatic mothers (19, 20), suggesting that maternal inheritance, including mitochondrial DNA (mtDNA), could contribute to asthma susceptibility.

Mitochondria are specialized organelles responsible for cellular energy production and are inherited exclusively from the mother. They contain their own genome, encoding key proteins and RNA molecules essential for oxidative phosphorylation (OXPHOS), which drives ATP production and supports cellular metabolic balance. In addition to generating energy, mitochondria play crucial roles in regulating calcium homeostasis, apoptosis, and the production of reactive oxygen species (ROS). Excessive ROS production can lead to oxidative stress and activate inflammatory pathways, contributing to chronic inflammation and tissue damage (21). Mitochondrial dysfunction, resulting from mutations or variants in mtDNA, can impair OXPHOS efficiency, leading to increased ROS levels and promoting inflammatory responses—central mechanisms implicated in asthma pathogenesis (22).

Previous studies have investigated the potential role of mitochondrial genetic background in asthma. Raby et al. (23) identified an association between mitochondrial haplogroup U and elevated total serum IgE levels in children with asthma, primarily in Caucasian populations. Similarly, Zifa et al. (24) reported specific mtDNA mutations more frequent in asthmatics than in controls in a Greek cohort. Studies conducted in Chinese (25) and German (26) populations also identified mtDNA variants associated with asthma risk or severity. More recently, studies in admixed and African ancestry populations, such as those by Vergara et al. (27) and Xu et al. (28), have reported associations between maternal African mitochondrial ancestry or mtDNA copy number and asthma-related traits. Collectively, these findings support the role of mitochondrial genetic variation in asthma susceptibility across multiple ancestries.

However, the specific contributions of mtDNA in Middle Eastern populations—particularly those with substantial African and Asian haplogroup components—remain largely unexplored. This study addresses this gap by investigating mitochondrial variation in a Kuwaiti cohort, providing novel insights into population-specific asthma risk mechanisms.

We previously demonstrated the effectiveness of next-generation sequencing (NGS) whole-exome data for capturing the mitochondrial genome. Our comparative analyses using duplicate samples sequenced via whole-genome and Sanger sequencing revealed high concordance in identifying mitochondrial variants and haplogroups, validating the utility of exome data for mitochondrial genetic analysis (29, 30).

Building on this background, we investigated mitochondrial DNA variation and haplogroup distribution in a Kuwaiti cohort to assess potential associations with asthma susceptibility.

## Materials and methods

### Ethical considerations

The study was conducted in accordance with the principles outlined in the Declaration of Helsinki (2008 amendments). Written informed consent was obtained from all participants, and ethical approval was granted by the Ethical Review Committee at Dasman Diabetes Institute. For further details on the recruitment process and initial cohort characteristics, please refer to John et al. (31).

### Study exome data

A total of 287 self-reported Kuwaiti participants, who were part of a population genetics study in Kuwait and selected to be free from rare genetic disorders, were divided into two groups: asthma (48 individuals) and control (239 individuals). All participants were adults (≥18 years) and provided written informed consent. Inclusion criteria included Kuwaiti nationality. Individuals with known monogenic or immune-related disorders were excluded, while those with common complex conditions were not, to reflect the general adult population (31). These individuals, described in detail in John et al. (31), underwent whole-exome sequencing using the Illumina HiSeq platform (Illumina Inc., USA) with two different capture kits: the TruSeq Exome Enrichment kit (*obsolete*) or its replacement the Nextera Rapid Capture Exome kit. The sequencing protocols, DNA extraction methods, and quality control measures are detailed in John et al. (31).

Variant-level data (including sample IDs, mitochondrial variants, and haplogroup classifications) for all individuals are provided in Supplementary Table S1. Raw sequencing data are publicly available under the Sequence Read Archive (SRA) accession number PRJNA1162699.

### mtDNA sequences, variants calling, annotation and haplogroups classification

Raw paired-end reads generated from whole-exome sequencing were aligned to the GRCh37 human genome assembly, which includes both nuclear and mitochondrial sequences, using BWA-MEM version 0.7.17 with default settings (32). This strategy reduces misalignment of nuclear mitochondrial DNA segments (NUMTs), as NUMT-derived reads preferentially align to their nuclear locations (33). Duplicate reads were removed using Picard tools version 2.20.2, and mtDNA sequences (NC_012920.1) were extracted using SAMtools version 0.1.19 (34). Coverage for the mitochondrial genome was calculated using SAMtools depth, yielding an average coverage of 27×, which was sufficient for reliable variant calling and haplogroup assignment (29, 30).

Variant calling was performed using the Genome Analysis Toolkit (GATK) version 3.8-1-0 (35) with the HaplotypeCaller module. The output was generated as Genomic Variant Call Format (GVCF) files, which were subsequently merged to produce a multi-sample Variant Calling Format (VCF) file. As this study focused on fixed mtDNA variants, the potential influence of residual NUMT reads is expected to be minimal.

Haplogroups were assigned using the HaploGrep2 tool (36). This was based on PhyloTree build 17 (accessed March 5, 2024), via upload of the mtDNA VCF file to the online platform. Annotation of the mtDNA variants was carried out using the Ensembl Variant Effect Predictor (37), incorporating functional predictions from SIFT and PolyPhen-2. Additionally, variants were cross-referenced with MitoMaster (38) and ClinVar (https://www.ncbi.nlm.nih.gov/clinvar/) databases to assess potential clinical relevance.

### Statistical analyses

Descriptive statistics for clinical characteristics were performed using R software version 3.6.2 (https://www.R-project.org/). Categorical variables, including sex and smoking status, were presented as numbers and percentages. In contrast, continuous variables, such as age and BMI, were presented as mean ± standard deviation (SD), median, and interquartile range (IQR). The Chi-square test was used to examine associations between categorical variables and asthma. Since age and BMI scores were not normally distributed, as determined by the Shapiro–Wilk test, the Mann–Whitney *U*-test was employed to evaluate their association with asthma.

### Principal component analysis (PCA)

To investigate potential hidden relationships and batch effects, PCA was performed using the complete set of mtDNA variants. The PCAtools package in R software version 3.6.2 was employed to generate biplots using the first and second principal components (PCs) to visualize clustering patterns and assess the influence of covariates.

### Association analysis

Nominal associations between asthma and mtDNA haplogroups were tested using Fisher's exact test. Odds ratios (OR) and 95% confidence intervals (CI) were calculated for each haplogroup, with a *P*-value < 0.05 considered statistically significant. To adjust for covariates (age, sex, and BMI), logistic regression was performed using IBM® SPSS® Statistics Version 25 software. The covariates age, sex, and BMI were selected to account for their influence on asthma risk within our cohort. Age was included due to its association with varying asthma prevalence (18), sex was adjusted for based on observed differences in susceptibility (2), and BMI was incorporated due to its significant association with asthma in our cohort (Table 1; *P* = 0.003) and link to inflammation (6). These were controlled for in the logistic regression to minimize confounding and isolate genetic associations with asthma. For mtDNA variant associations, logistic regression was implemented using PLINK version 1.9 (39), adjusting for age, sex, BMI, and haplogroup to minimize confounding effects. Haplogroup adjustment accounted for potential maternal lineage effects, addressing population structure to ensure reliable genetic associations. Multiple testing corrections, such as Bonferroni or Benjamini-Hochberg procedures, were not applied, reflecting the exploratory nature of the analysis and the complexity of mitochondrial DNA, given that asthma is a complex, multifactorial disease where these associations may involve cumulative variant effects.

**Table 1:** Clinical characteristics of the Kuwaiti asthma study.

	Asthmatic (*N* = 48) *n* (%)	Control (*N* = 239) *n* (%)	Total (*N* = 287) n(%)	*P*-value
Sex				
Male	19 (40%)	98 (41%)	117 (41%)	0.98
Female	29 (60%)	141 (59%)	170 (59%)	
Age (years)				
≤50	16 (33.3%)	119 (49.8%)	135 (47%)	0.08
>50	32 (66.7%)	120 (50.2%)	152 (53%)	
Mean ± SD	51.7 ± 10.9	53.7 ± 10.9	52.5 ± 11	
Median (IQ)	51 (44–59)	54 (47–60)	52.5 (45–59)	
Smoking				
Yes	7 (14.6%)	52 (22.8%)	59 (20.6%)	0.35
No	41 (85.4%)	187 (78.2%)	228 (79.4%)	
BMI score				
Mean ± SD	36.4 (10.3)	31.8 (8.7)	32.6 ± 9.1	0.003
Median (IQ)	35.4 (29.1–42)	30.1 (24.6–38.3)	31(25.3–38.9)	

*P*-values for continuous age and BMI scores were calculated using the Mann–Whitney *U*-test. *P*-values for sex and smoking status were calculated using the Chi-square test.

To address potential inflation due to case-control imbalance, we performed a sensitivity analysis using Firth logistic regression in PLINK v2.0 with the –glm firth-fallback option, using the same covariates as the primary analysis. This method applies penalized likelihood estimation to reduce bias in unbalanced datasets.

### Exclusivity analysis

Exclusivity analysis was conducted to identify mtDNA variants recurrently present in asthmatic individuals, thereby increasing the likelihood of disease association. Variants were retained only if they were detected in more than one asthma patient and completely absent from the control group. Variants present in any control or found in only one asthmatic individual were excluded. The retained variants were then subjected to Fisher's exact test, with a *P*-value < 0.05 considered significant.

## Results

### Study population

The descriptive statistics for the dataset, comprising 287 Kuwaiti individuals, are presented in Table 1. The study population included 48 asthmatic individuals and 239 controls. There were no significant differences between cases and controls regarding sex, age, or smoking status. However, the results of the Mann–Whitney *U*-test for BMI scores showed that individuals with asthma had significantly higher BMI scores compared to control samples (*P* = 0.003).

### mtDNA coverage and variants

To assess the uniformity of mitochondrial genome coverage across the 287 samples analyzed, we generated a genome-wide coverage profile from the WES data. As shown in Supplementary Figure S1, the coverage was consistent across nearly all mtDNA positions, with an average depth of ∼27× and no evidence of regional dropout.

### Principal component analysis (PCA)

**Figure 1:**
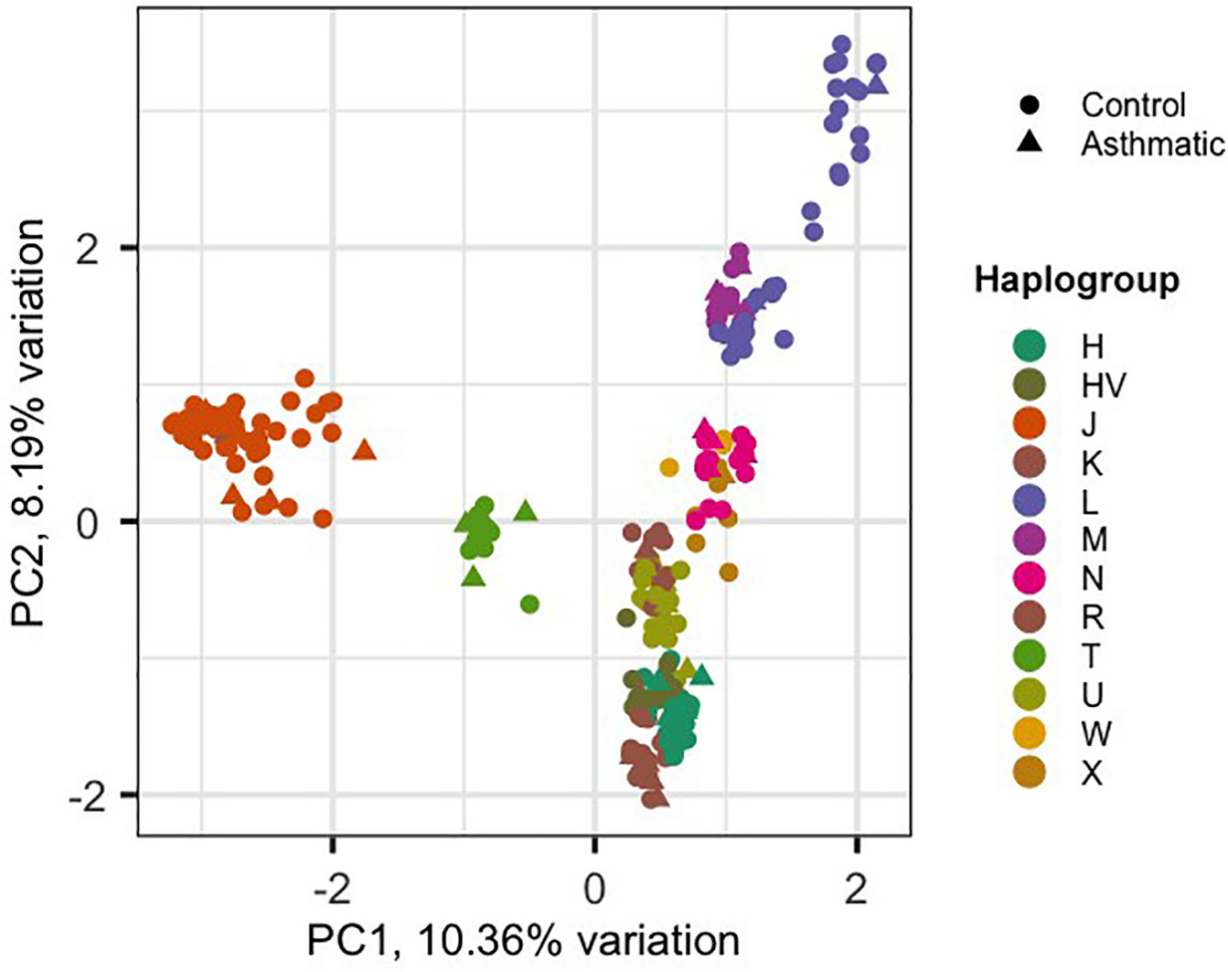
PCA analysis of 287 Kuwaiti samples based on their mtDNA.

Using the HaploGrep 2 tool, we identified 12 mitochondrial haplogroups (H, HV, J, K, L, M, N, R, T, U, W, X) across the sample set. Among these, haplogroup J was the most prevalent across the entire sample set (*n* = 287; 54 individuals, as shown in Table 2). The average quality score of predicted haplogroups for all samples was above 91%, indicating high accuracy in haplogroup assignment. Principal component analysis (PCA) was performed on 1,241 mitochondrial variants from 287 Kuwaiti samples (48 asthmatic, 239 controls). PCA revealed clustering patterns consistent with haplogroup assignment, indicating that mtDNA variants could capture maternal lineage without confounding effects. The biplot of PC1 vs. PC2 (Figure 1) shows the distribution of samples based on their haplogroups, confirming no significant population stratification.

**Table 2:** Mitochondrial haplogroup associated with asthma in the Kuwaiti population.

Haplogroups	Asthmatic N (48)	Control N (239)	OR	*P*-value	OR (95% CI)*	*P*-value*
H	10 (20.83%)	36 (15.06%)	1.48	0.32	1.553 (0.696–3.468)	0.283
HV	0 (0%)	10 (4.18%)	0	0.149	–	0.997
J	5 (10.41%)	49 (20.50%)	0.45	0.103	0.423 (0.156–1.152)	0.092
K	2 (4.16%)	8 (3.34%)	1.26	0.778	1.190 (0.229–6.176)	0.836
L	3 (6.25%)	33 (13.80%)	0.42	0.149	0.337 (0.095–1.193)	0.092
**M**	**6** (**12.5%)**	**9** (**3.76%)**	**3**.**65**	**0**.**013**	**3.371** (**1.091–10.416)**	**0**.**035**
N	4 (8.33%)	17 (7.11%)	1.19	0.767	1.277 (0.395–4.126)	0.683
R	7 (14.58%)	25 (10.46%)	1.46	0.408	1.696 (0.666–4.321)	0.268
T	5 (10.41%)	14 (5.85%)	1.87	0.246	1.556 (0.511–4.742)	0.436
U	5 (10.41%)	27 (11.29%)	0.91	0.86	1.098 (0.387–3.118)	0.86
W	0 (0%)	3 (1.25%)	0	0.435	–	0.998
X	1 (2.08%)	8 (3.34%)	0.61	0.647	0.631 (0.075–5.332)	0.672

Bold values indicate statistically significant associations between mitochondrial haplogroups and asthma in the Kuwaiti population (*P* < 0.05).

* Values after adjustment for age, sex, and BMI.

N, number of individuals; OR, odds ratio; CI, confidence intervals.

The control group, consisting of individuals without asthma, is represented by circles, while the case group, consisting of individuals with asthma, is represented by triangles. The *x*-axis and *y*-axis denote the first and second principal components (PC1 and PC2), respectively, with the percentage of variation they explain.

### Mitochondrial haplogroups association with asthma

Haplogroup association analysis revealed that individuals with haplogroup M had a significantly higher risk of developing asthma (OR = 3.65; *P* = 0.013). After adjusting for age, sex, and BMI, the association remained significant (OR = 3.37; 95% CI = 1.091–10.416; *P* = 0.035). Asthma is a complex, multifactorial disease, and this association may reflect cumulative effects of variants within haplogroup M, potentially interacting with environmental factors. This *P*-value would not remain significant after correction for the multiple haplogroups tested (*n* = 12), consistent with the exploratory approach given the numerous mtDNA variants identified.

### Mitochondrial variant association with asthma

In total, nine mitochondrial variants were found to be significantly associated with asthma (*P*-values <0.05) using univariate analysis (Table 3). Of these, eight variants remained significant after adjusting for age, sex, BMI, and maternal haplogroups using multivariate logistic regression. All eight variants were positively associated with asthma (OR > 1). The one variant that lost its significance after adjustment was an upstream variant in the *MT-TP*, specifically MT:16519C > T, which was negatively associated with asthma (OR < 1). Another upstream variant in the *MT-TP* gene, MT:16359T > C, had the most significant *P*-values (0.015 in univariate and 0.005 in multivariate analyses), with a 15-fold and 27-fold likelihood of being associated with asthma, respectively.

**Table 3:** Mitochondrial variants associated with asthma in the Kuwaiti population.

mtDNA Variant	Gene	Consequence	Asthmatic F	Control F	OR (95% CI)	*P*-value	OR (95% CI)*	*P*-value *
MT:16359T > C	*MT-TP*	Upstream	0.062	0.004	15.8 (1.607–155.3)	0.015	27.8 (2.682–288.3)	0.005
MT:7853G > A	*MT-CO2*	Missense	0.062	0.004	15.73 (1.6–154.7)	0.015	18.25 (1.69–197.1)	0.016
MT:14783T > C	*MT-CYB*	Synonymous	0.125	0.037	3.61 (1.224–10.7)	0.025	3.52 (1.143–10.84)	0.028
MT:10400C > T	*MT-ND3*	Synonymous	0.125	0.038	3.57 (1.208–10.56)	0.026	3.47 (1.127–10.72)	0.030
MT:15043G > A	*MT-CYB*	Synonymous	0.130	0.042	3.40 (1.172–9.892)	0.029	3.21 (1.057–9.8)	0.039
MT:12403C > T	*MT-ND5*	Missense	0.062	0.008	7.9 (1.283–48.63)	0.034	8.49 (1.239–58.27)	0.029
MT:16248C > T	*MT-TP*	Upstream	0.062	0.008	7.86 (1.278–48.42)	0.034	7.30 (1.119–47.64)	0.037
MT:14110T > C	*MT-ND5*	Missense	0.062	0.008	7.83 (1.273–48.22)	0.035	8.43 (1.232–57.76)	0.029
MT:6446G > A	*MT-CO1*	Synonymous	0.062	0.008	7.76 (1.262–47.81)	0.035	8.49 (1.234–58.49)	0.029
MT:16519C > T	*MT-TP*	Upstream	0.266	0.437	0.46 (0.230–0.952)	0.045	0.51 (0.243–1.086)	0.081

* Values after adjustment for age, sex, BMI, and mitochondrial haplogroup.

F, frequency; OR, odds ratio; CI, confidence intervals.

Post hoc power analysis was conducted to evaluate the study's ability to detect observed associations. For haplogroup M (12.5% in cases vs. 3.76% in controls), power estimates ranged from 56% (two-proportion z-test) to 68% (chi-squared method). For the most significant variant, MT:16359T > C (6.2% in cases vs. 0.4% in controls), the estimated power was approximately 66%, indicating moderate statistical power. To account for potential inflation due to case-control imbalance, we performed a sensitivity analysis using Firth logistic regression (Supplementary Table S2). All mitochondrial variants that were significant in the standard logistic regression remained significant, and none required penalization (FIRTH? = “No”), suggesting that these associations are unlikely to be driven by imbalance-related bias.

**Figure 2:**
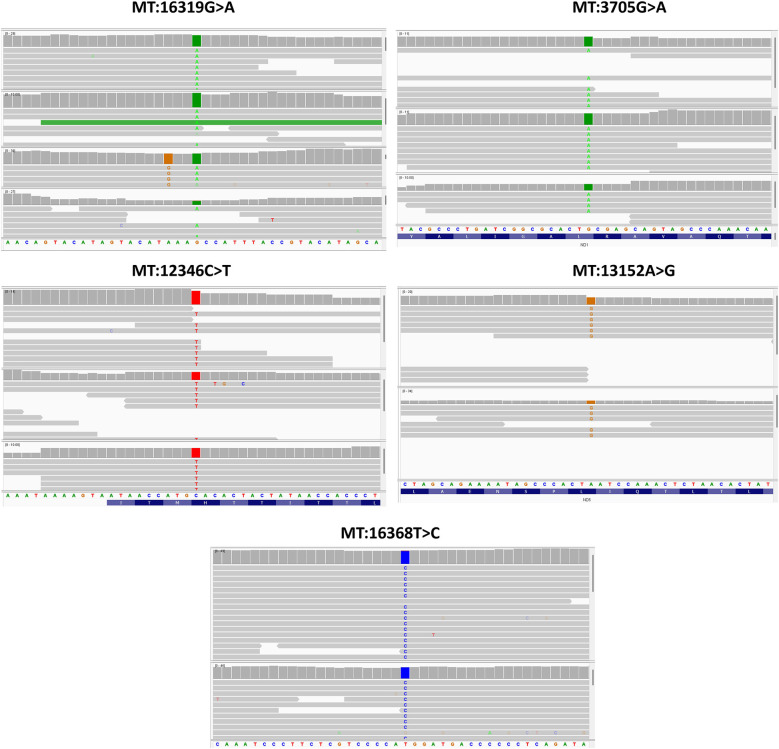
IGV visualization of the five low-frequency mtDNA variants identified exclusively in asthma patients.

Moreover, five SNPs were detected exclusively in the asthmatic group, in at least two individuals each (Table 4). These SNPs were also found to be significant when analysed using Fisher's exact test. Among them were two upstream variants, MT:16319G > A and MT:16368T > C, in the *MT-TP* gene. Additionally, two coding variants in the *MT-ND5* gene [nicotinamide adenine dinucleotide (NADH) dehydrogenase subunit 5]—a missense variant (MT:12346C > T) and a synonymous variant (MT:13152A > G)—and one synonymous variant in the *MT-ND1* gene (NADH dehydrogenase subunit 1) (MT:3705G > A) were identified. To further evaluate the reliability of these low-frequency mtDNA variants, we performed manual inspection using the Integrative Genomics Viewer [IGV; (40)]. All variants were visually confirmed with clear read support (Figure 2).

**Table 4:** Significant associated mtDNA variants that present only in asthmatic individuals (>1).

mtDNA variants	Gene	Consequence	Number of individuals	Amino acids	SIFT	PolyPhen	*P*-value
MT:16319G > A	*MT-TP*	Upstream	4	–	–	–	0.001
MT:3705G > A	*MT-ND1*	Synonymous	3	L	–	–	0.004
MT:12346C > T	*MT-ND5*	Missense	3	H/Y	tolerated	unknown	0.004
MT:13152A > G	*MT-ND5*	Synonymous	2	L	–	–	0.027
MT:16368T > C	*MT-TP*	Upstream	2	–	–	–	0.028

The *in silico* functional analysis using SIFT and PolyPhen-2 tools suggested that none of the mtDNA exonic variants were predicted to be pathogenic or likely pathogenic. However, the clinical significance of the synonymous variants MT:14783T > C and MT:15043G > A in the *MT-CYB* gene, both positively correlated with asthma, has been reported as likely pathogenic for familial breast cancer in the ClinVar database. Additionally, the Mitomaster database indicates that the MT:15043G > A variant is associated with Major Depressive Disorder (41). Furthermore, the synonymous variant MT:10400C > T, which is positively correlated with asthma, has been associated with gastric cancer susceptibility (42) and Leber hereditary optic neuropathy (43) in the literature.

IGV screenshots showing read alignments at the genomic positions of the five mtDNA variants listed in Table 4. Each panel highlights the variant position, where coloured bases represent mismatches from the reference genome. The alignments show consistent read support from both strands, along with depth and base quality metrics, confirming the presence of these variants despite their low allele frequency in the cohort.

## Discussion

Kuwait's unique genetic and environmental context provides a window into how mtDNA haplogroups and variants can influence asthma. The 1991 oil fires and ongoing pollution have imposed chronic oxidative stress on the population (44–46). These environmental stressors likely interact with genetic factors –particularly mitochondrial variants– to modulate OXPHOS efficiency and inflammatory responses (21, 22).

In this context, we investigated the association between mtDNA haplogroups and variants with asthma risk by analysing indirect whole-exome sequences from 287 Kuwaiti individuals, with an average mtDNA coverage of 27× Principal component analysis revealed that individuals sharing the same maternal haplogroup clustered together, supporting the accuracy of our haplogroup profiling and indicating minimal confounding factors.

The maternal haplogroup association analysis revealed mitochondrial haplogroup M as a significant risk factor for asthma in the Kuwaiti population. Individuals in our study carrying maternal haplogroup M had a threefold increased risk of developing asthma (OR = 3.371; 95% CI = 1.091–10.416; *P* = 0.035) after adjusting for covariates. To our knowledge, this is the first study to report such an association. Haplogroup M is predominantly found in Asian and African populations compared to European populations (47) and may confer asthma risk due to specific mitochondrial variants that contribute to mitochondrial dysfunction, oxidative stress, and inflammation—pathways strongly implicated in asthma pathogenesis (22).

Kuwait's population has a distinctive genetic makeup shaped by admixture from settlers of Saudi Arabian, Iranian, and other Arabian Peninsula origins, with a significant African contribution observed among its Bedouin subgroups (48). Thus, the identification of haplogroup M as an asthma risk factor in this study likely reflects unique genetic backgrounds interacting with specific regional environmental exposures, such as elevated pollution levels and allergen prevalence.

Our finding contrasts notably with previous studies focusing primarily on European-ancestry cohorts, where haplogroup U rather than M has been implicated. For instance, Raby et al. (23) observed that haplogroup U carriers in North America exhibited significantly higher serum IgE levels and greater atopic sensitization compared to non-carriers. Similarly, Zifa et al. (24) found a significant association between haplogroup U and asthma severity in a Greek cohort, where 27.6% of asthma patients belonged to haplogroup U compared to only 4% of controls. These earlier studies suggest a mitochondrial maternal lineage effect underlying asthma susceptibility and severity. In our Kuwaiti cohort, haplogroup U showed no significant association with asthma, nor did it follow frequency trends previously observed in Western populations; its frequency was marginally higher (by approximately 1%) in controls compared to asthmatics. This discrepancy highlights the complexity of mitochondrial genetic factors in asthma and emphasizes the importance of investigating diverse populations. While genetic background and environmental context may contribute to population-specific associations, the lack of replication of previously reported haplogroup U associations may also reflect limited statistical power in our modest cohort. Therefore, the novel association between haplogroup M and asthma identified here likely arises from mitochondrial polymorphisms within haplogroup M sub-clades that may confer neutrality or selective advantage in their original geographic context but predispose carriers to asthma in Kuwait's specific environmental conditions.

Beyond haplogroup comparisons, we identified 10 exonic mitochondrial variants associated with asthma risk through two complementary analyses: case-control association testing (Table 3) and exclusivity analysis (Table 4), within genes encoding subunits of Complex I (*MT-ND1*, *MT-ND3*, *MT-ND5*), Complex III (Cytochrome b gene, *MT-CYB*), Complex IV (Cytochrome c oxidase genes, *MT-CO1*, *MT-CO2*), and the D-loop region upstream of the mitochondrial tRNA-Pro (*MT-TP*) gene. Complex I mediates electron transfer from NADH to ubiquinone, Complex III facilitates electron transfer from ubiquinol to cytochrome c, and Complex IV catalyses electron transfer to molecular oxygen. Genetic variants within these complexes can disrupt mitochondrial electron transport, resulting in mitochondrial dysfunction, elevated ROS production, oxidative stress, and inflammatory responses—key mechanisms underlying asthma pathogenesis (49–53). Notably, both missense and synonymous variants were identified in these critical genes. Although our study did not directly assess functional impact, prior research has shown that similar types of variants in these same mitochondrial genes can disrupt mRNA stability, translation, or electron transport, potentially leading to excess ROS and inflammation (49, 54, 55). These mechanisms may underlie the biological relevance of the observed associations in asthma. Previous research by Fukuda et al. (56) identified altered expression of mitochondrial respiratory chain genes, specifically subunits of Complex I (NADH dehydrogenase) and Complex IV (Cytochrome c oxidase II and III), in allergic airway conditions, collectively reinforcing the relevance of mitochondrial genetic variants in asthma susceptibility.

In addition to exonic variants, our study identified variants in the mitochondrial control region (MT:16248C > T, MT:16319G > A) and upstream of the tRNA-Pro gene (MT:16359T > C) associated with asthma susceptibility. The control region variants (MT:16248C > T, MT:16319G > A), located in hypervariable region 1 (HVR1), are traditionally linked to haplogroup-defining polymorphisms but may also influence mtDNA replication or transcription efficiency. Alterations in control region activity could modulate mitochondrial copy number or nucleoid organization, indirectly exacerbating oxidative stress in airway cells—a mechanism implicated in asthma pathogenesis (28). While the upstream MT:16359T > C variant does not directly alter the tRNA-Pro gene, previous studies have demonstrated that mitochondrial tRNA mutations are significantly more frequent in asthmatic patients than in controls (24–26). Although our study did not detect the MT:3243A > G mutation in tRNA-Leu(UUR)—a variant classically linked to MELAS syndrome but reported in other asthma cohorts (57)—the observed enrichment of tRNA-Pro variants in our cohort supports the broader role of mitochondrial tRNA destabilization in airway disease. Such tRNA defects impair respiratory chain biogenesis, elevate ROS production, and amplify inflammatory responses in airway epithelia (52). Interestingly, one *MT-TP* variant (MT:16519C > T) initially exhibited a protective effect against asthma in our cohort (OR = 0.46; *P* = 0.045); however, this association did not remain significant after adjusting for covariates. This suggests that the protective effect may be influenced by confounding factors, warranting further investigation.

While SIFT and PolyPhen-2 predicted low pathogenicity for the variants identified in our study, these tools were developed for nuclear genes and may not reliably assess mitochondrial variants. Wang et al. (58) reported only ∼57% concordance between predictions and known mtDNA variant classifications, highlighting the need for cautious interpretation of such results. Moreover, ClinVar does not annotate any mtDNA variants for asthma, including those identified here, across all clinical classifications. However, some are linked to other conditions sharing mitochondrial dysfunction, inflammation, or oxidative stress with asthma pathogenesis (59). For example, familial breast cancer and gastric cancer involve chronic inflammation and ROS overproduction (60, 61); major depressive disorder exhibits comorbidity with asthma via cytokine dysregulation (62); and Leber hereditary optic neuropathy features mitochondrial impairment with elevated ROS (63), akin to airway oxidative stress in asthma.

This study has certain constraints that warrant acknowledgment. The primary limitation is the modest cohort size (*n* = 287, including 48 asthmatic individuals), which limits the statistical power to detect associations with rare mitochondrial haplogroups or low-frequency variants. *Post hoc* power analysis indicated moderate power for key findings (56%–68% for the haplogroup M association and ∼66% for representative mtDNA variants), highlighting the need for replication in larger, independent cohorts. The retrospective nature of the study and its nesting within a broader population-based genetic cohort resulted in a fixed case-control ratio. To address potential bias from this imbalance, we performed a sensitivity analysis using Firth logistic regression, which showed that the primary associations remained statistically significant. Additionally, asthma status was self-reported, precluding stratification by disease severity or immunological subtypes (e.g., eosinophilic asthma) and limiting adjustment for clinical covariates such as IgE levels. While WES is not primarily designed for mtDNA capture, previous validations—including our own—support its utility in detecting homoplasmic variants and assigning haplogroups. Our analysis showed consistent coverage across the mitochondrial genome (mean depth ∼27×) without regional dropout; meeting depth thresholds commonly used for reliable variant calling. However, this depth is suboptimal for detecting low-frequency heteroplasmic variants, which require higher coverage and targeted sequencing to distinguish from technical artifacts (33). Heteroplasmy is also tissue-specific; thus, blood-derived mtDNA may not fully represent disease-relevant mitochondrial variation in airway tissues (64). Multiple testing correction was not applied, as conventional methods (e.g., Bonferroni) can be overly conservative for mitochondrial data due to its linked, non-recombining structure, potentially masking cumulative effects in a multifactorial disease like asthma. Future studies should incorporate larger cohorts, deeper tissue-targeted sequencing, and orthogonal validation methods to more accurately characterize mtDNA heterogeneity in asthma.

## Conclusion

Despite its limitations, this study provides novel insights into the role of mitochondrial genetics in asthma susceptibility within the Kuwaiti population. We identified mitochondrial haplogroup M as a significant risk factor, along with several mtDNA variants in genes directly involved in mitochondrial dysfunction and ROS production—both of which are implicated in asthma pathogenesis. These findings emphasize the interplay between maternal lineage, mitochondrial biology, and asthma risk, offering a foundation for future research. Further functional studies—including *in vitro* or *in vivo* experiments—in larger Middle Eastern cohorts are necessary to validate these associations and to explore how mitochondrial genetic profiles interact with environmental stressors to influence asthma outcomes.

## Data Availability

The original contributions presented in the study are included in the article/Supplementary Material, further inquiries can be directed to the corresponding author/s.
