# Supplementary figures and images for: Mitochondrial haplogroup M contributes to asthma risk in the Kuwaiti population
Source: Front Allergy. 2025 Aug 8;6:1618964. doi: 10.3389/falgy.2025.1618964 (PMC12370696; doi:10.3389/falgy.2025.1618964)

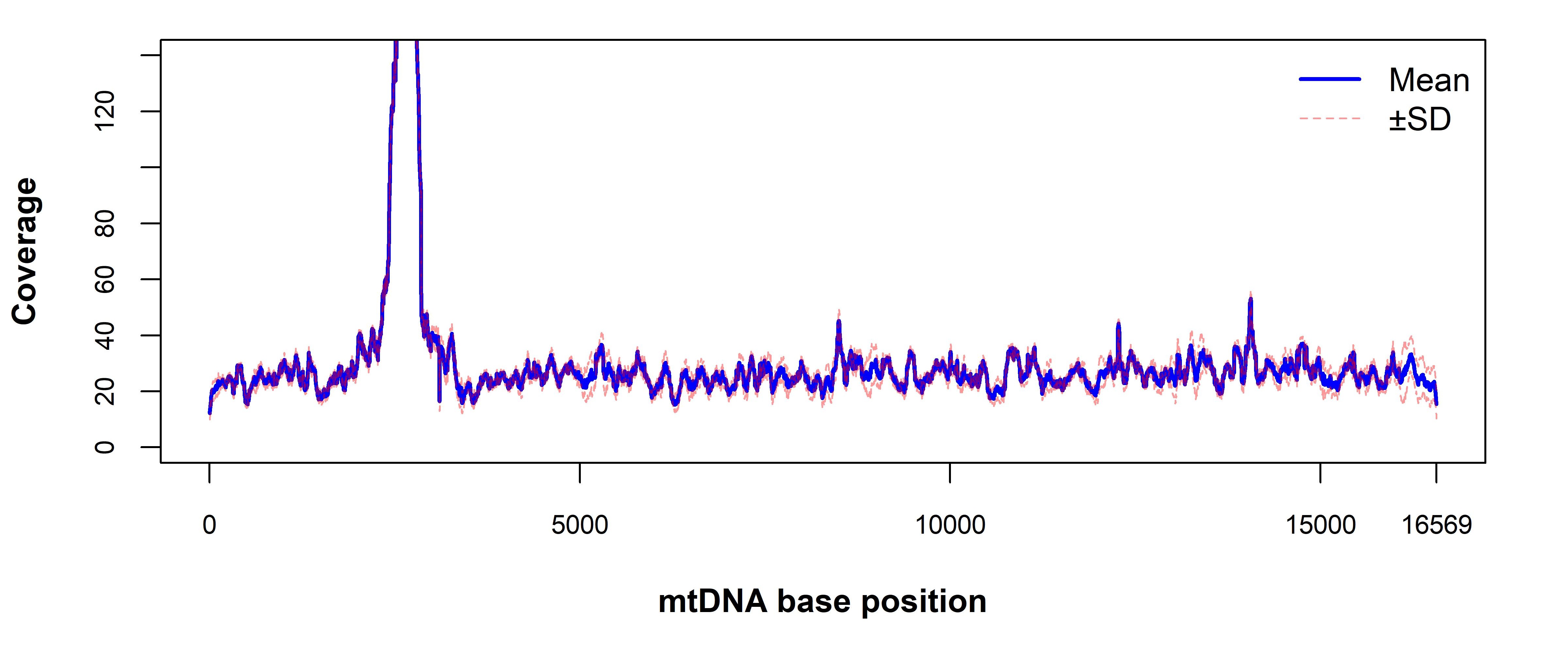

Supplement: Supplementary file 3 [file Image1.jpeg]
